# Green practices and economic performance: Mediating role of green innovation in Ethiopian leather, textile, and garment industries—An integrated PLS-SEM analysis

**DOI:** 10.1016/j.heliyon.2024.e35188

**Published:** 2024-07-25

**Authors:** Tilahun Nigatu, Aschalew Degoma, Abiot Tsegaye

**Affiliations:** a Department of Management, Arba Minch University, Ethiopia; b Department of Management, Bahir Dar University, Ethiopia

**Keywords:** Green marketing orientation, Green manufacturing, Green innovation, Green HRM, Green investment, Structural equation model, Economic performance

## Abstract

Incorporating green issues into business operations is indisputable, as this is mandatory in today's environmental era. The purpose of this study was to explore the structural link between green practices such as green manufacturing, green marketing orientation, green human resource management, green investment, and the economic performance of textile and leather firms in Ethiopia through green innovation. This study was carried out utilizing a quantitative method, and 201 surveys were used to gather the data from the top managers and general managers of medium and large textile and leather firms in Ethiopia using stratified random sampling. To test the hypothesis, partial least squares structural equation modeling (PLS-SEM) was used. The results of this study demonstrate that green practices, specifically green manufacturing practices (GMP), green marketing orientation (GMO), and green investment (GIV), have a positive and substantial contribution to firms' economic performance (EP) and green innovation (GIN). However, the advancement of GIN is not significantly affected by the adoption of sustainable human resources management (HRM). Moreover, green innovation facilitates the connection between economic success, GIV, and GMO. Nonetheless, when it comes to the relationship between GHRM, green manufacturing practices, and economic success, green innovation plays no mediating function. According to this research, a company's management ought to strengthen its green practices as lower expenses would boost its economic outcome.

## 1 Introduction

Companies today employ green initiatives to efficiently generate and provide value to their customers [1]. Eco-friendly practices are also necessary for sustainability, which is a difficult task because of the environmental and socioeconomic problems that irresponsible business practices create [2]. Sustainable business practices are a collection of tactics and programs designed to incorporate sustainability ideas into company operations [1]. Green investment practices, green marketing strategies, green manufacturing techniques, and green HRM are some of these activities [3]. Utilizing eco-friendly manufacturing practices, for example, reducing waste, using green power sources, and optimizing resource use is known as “green manufacturing” [4]. Promoting employee participation, education, and awareness is the main goal of green HRM to cultivate a sustainable culture inside the company [5]. A crucial aspect of the ecological marketing approach is the creation and promotion of ecologically friendly products and services in response to consumer demands for sustainability [6]. Allocating funds for eco-friendly projects, R&D, and sustainable technology is what's known as the “green investment” technique [7,8]. Furthermore, the creation and use of novel concepts, procedures, and goods that support environmental sustainability is known as “green innovation” [9,10]. It is essential in helping companies balance environmental responsibility with economic success [11]. Green innovation serves as a channel via which ecologically sensitive company activities are translated into observable economic benefits by moderating the association between sustainability business practices and economic outcomes [12].

Recently, Ethiopia's leather and textile industries have grown dramatically, making an outstanding impact on the economic development of the nation [13]. Concerns over these industries' effects on the environment have also been raised by their rise [14]. As a result, companies operating in the leather and textile industries are progressively using environmentally friendly business practices to reduce their environmental impact while achieving profitability [15]. However, there are some misconceptions regarding the association amongst eco-friendly activities and financial results [16], especially in developing economies like Ethiopia. For example, some people think that adopting green practices will be detrimental, especially in developing economies [17] like Ethiopia. A dearth of knowledge about the possible financial advantages of environmentally friendly practices could also result from a lack of exposure to success stories and actual data relevant to Ethiopia. Additionally, there is a lack of thorough investigations on the synergistic impact of green procedures on economic outcomes, which creates a substantial research vacuum in emerging economies like Ethiopia. Previous research has predominantly concentrated on developed economies, resulting in a scarcity of studies [18]. In emerging economies like Ethiopia, to profit economically from green practices and to encourage their implementation, it is imperative to dispel these myths.

Thorough studies on the synergistic effects of green activities on economic performance can offer insightful details and supporting data to clarify these myths and emphasize the possible benefits of adopting green procedures. To this end, the underlying link between green business techniques—like green manufacturing, green HRM, Eco-friendly investing practices, and marketing strategies —and the financial success of Ethiopia's leather and textile sectors constitutes the primary focus of this study. Moreover, investigating environmentally friendly inventions mediation role in this connection is of special emphasis.

The research investigates the connection between Ethiopia's leather, textile, and apparel sectors' financial success and green practices. It provides a thorough grasp of the connection between green practices, innovation, and economic success using a variety of theoretical frameworks. The study adds to the body of knowledge on sustainability in developing nations by concentrating on the Ethiopian setting. The results can help guide policymakers in promoting green practices and innovation in various industries and can also be used to assist management decision-making. For more extensive applications in reality, the theoretical basis and analytical methodology ought to be extended to additional emerging nations and industries.

## 2 Literature review

Environmentally friendly company practices are a broad category of practices and programs designed to mitigate the negative effects of corporate practices on the environment while fostering sustainability [1]. Green manufacturing, green marketing strategies, green HRM practices, green investment techniques, and green innovation are some of these approaches [3].

Specifically, using eco-friendly methods throughout the production process is the main goal of green manufacturing [19]. This entails employing recyclable materials, cutting pollution and wasteful activities, and maximizing the efficiency of energy and resources [20]. To reduce their environmental impact, industries can use lean manufacturing techniques, make investments in renewable energy sources, and switch to greener methods of production [1].

Including environmental issues in a business's marketing plans and endeavors is known as “green marketing orientation” [21]. Promoting environmentally friendly goods and services, highlighting their advantages for the environment, and informing customers of their beneficial effects on the environment are all part of it [6]. Showcasing qualities like recyclable materials, energy efficiency, sustainable sourcing, and lower carbon footprints is a common practice in green marketing [22]. Firms may draw in environmentally sensitive customers, set themselves apart from rivals, and develop a strong brand identity linked to sustainability by embracing a green marketing strategy [23].

Embracing environmental sustainability concepts in human resource management (HRM) inside a business is known as “green HRM” [24]. This covers actions like encouraging staff members to participate in environmental projects, offering instruction on sustainable practices, and incorporating environmentally friendly practices into rewards and performance reviews [25]. In addition, green HRM practices enhance achieving a harmonious work-life equilibrium and employee welfare, encourage employee participation in CSR projects, and cultivate a culture of sustainability [26]. Businesses may increase employee happiness, draw in and keep environmentally conscious personnel, and reaffirm their commitment to sustainability by using green HRM practices.

Putting money into ecologically friendly projects and activities is known as the “green investment” approach [27]. Investing in power-efficient technology, resilient infrastructure, clean energy sources, and eco-friendly goods and services are all included in this [28]. Organizations may help with the shift to a zero-carbon economy, promote innovation, and provide long-term financial benefits while tackling environmental issues by allocating resources to green businesses [29].

Green innovations are the creation and uptake of green goods, services, and processes that lessen their adverse impacts on ecological balance and advance sustainability [11]. This might entail developing new tools, procedures, and corporate strategies to promote eliminating waste, fuel efficiency, and the utilization of sustainable sources of power [30].

A firm's economic performance (EP) is often assessed using operational and financial results, with manufacturing enterprises potentially reducing charges pertaining to materials, waste management, energy usage, and penalties for violations of an ecological catastrophe [31]. Economic performance in manufacturing industries is determined by factors including financial metrics such as profits, income growth, market expansion, and returns on assets, equity, and investment [32]. The facility's capacity to cut expenses related to the acquisition of supplies, power use, waste management, sewage expulsion, and environmental fines is crucial for its success [33,34].

### 2.1 Empirical review and hypothesis

The empirical literature on the relationship between green business practices such as green manufacturing practice, green marketing orientation, green human resource management-training and development, and green investment with the economic outcome of leather, textile, and garment in Ethiopia and the mediating role of green innovation ware assessed as follows.

### 2.2 Green business practices and economic performance

#### 2.2.1 Green manufacturing practices and economic performance

Using eco-friendly procedures in manufacturing processes is known as “green manufacturing practice (GMP), and it may greatly boost a company's bottom line [35]. GMP reduces costs associated with the use of raw materials usage, energy consumption, removing waste, and complying with regulations by maximizing resource utilization, limiting waste creation, and increasing energy efficiency [36]. Lower manufacturing costs and better economic performance are the outcomes of this. Purchasing eco-friendly machinery and procedures increases productivity overall, decreases production bottlenecks, and improves the efficiency of operation [37]. GMP additionally enhances a company's economic performance [38]. While companies that wish to implement green manufacturing processes must pay upfront expenses, they will almost certainly reap benefits in the medium to long run [39] made the case that using green manufacturing techniques increases a company's market share and earnings. In favor of this, Çankaya and Sezen [40], have revealed in an investigation conducted among Turkish manufacturing enterprises that green manufacturing practices influence economic performance in addition to social and environmental performance. According to recent research in 401 large manufacturing organizations in Pakistan, implementing green manufacturing greatly enhances a firm's capability to generate revenue by lowering production costs and streamlining the workflow [41].

#### 2.2.2 Green marketing orientation and economic performance

Numerous empirical research has looked at the link between financial achievement and a green marketing perspective. The firm's priority on incorporating environmentally friendly practices into its marketing procedures and operations is known as “green marketing orientation” [42]. Attracting eco-aware customers, building brand equity, and eventually achieving profitable results are the goals [6].

The study indicates a favorable correlation between an emphasis on sustainable marketing and economic outcomes. As an illustration, Mishra et al. [43], study discovered that businesses that implemented green marketing strategies saw gains in market share and revenue, as well as enhanced consumer loyalty and brand perception. Peattie & Crane [21], research also showed that companies with a significant focus on green marketing did better financially than their rivals. Furthermore, Negi et al. [44], asserted that incorporating green issues in the firm's marketing operations can lead to an improving their economic performance.

#### 2.2.3 Green HRM and economic performance

To accomplish corporate goals, green HR methods guarantee the implementation of conventional HR procedures such as hiring, education, managing performance, and so on with an emphasis on ecological factors. Adopting green HR practices gives the company a long-term competitive edge [45], improves the company's standing [46], and boosts its profitability [47]. Research by Renwick et al. [26], and Zaid, Jaaron, & Talib Bon [48] revealed that combining green HR practices may constitute substantial consequences on an organization's success. To create a cumulative effect on the business, they offered a package of five greener HR procedures including hiring and selecting procedures, training and development procedures, task management procedures, and green remuneration [49]. When paired with other complimentary HR practices, this strategy may greatly improve the economic, social, environmental, and company outcomes [50]. According to the empirical evidence, implementing a bundle of green HR practices may greatly enhance corporate performance [51]. It has been discovered that using green human resource management (GHRM) techniques improves sustainability performance across a range of businesses [52]. Research indicates that green HR practices including green performance reviews, development, and training—are essential for improving sustainability [53]. Furthermore, as determined by the Triple Bottom Lines, the use of GHRM-training and development techniques have been connected to enhanced social, environmental, and economic performance [54]. Additionally, according to research, green human resource management (GHRM) techniques greatly improve Pakistani businesses' sustainability and environmental performance [55]. Research carried out by Ullah et al. [56], demonstrated the beneficial effects of greenhouse management practices (GHRM) on sustainable behavior and sustainability of organizations Moreover, studies highlight how crucial GHRM is to improving environmental performance through strategies like green engagement and psychological atmosphere that is supportive of the environment, which eventually improves the organization's monetary outcome [57].

#### 2.2.4 Green investment and economic performance

Studies indicated a favorable link between green investments and economic outcomes, as companies that make investments in sustainability enhancements and pollution reduction have greater market values and profitability [58]. A meta-analysis encompassing 52 research demonstrated a favorable correlation between financial success and environmental responsibility, suggesting that companies that prioritize environmentally conscious investments typically get superior economic outcomes [59]. Adopting energy-efficient technology, maximizing resource use, and minimizing waste creation are common practices in green investing, which result in lower costs and increased effectiveness [60]. Additionally, it might encourage inventiveness, resulting in the fabrication of new goods, services, and procedures to meet the rising need for eco-friendly solutions [61]. Businesses may boost market share and revenue growth, establish a competitive edge, and take advantage of market possibilities by tackling environmental concerns. Additionally, green investments may improve a company's standing with stakeholders, investors, and environmentally sensitive consumers. This can draw in new clients and improve ties with authorities. Because of lower expenses, greater productivity, potential for sales, adherence to regulations, risk reduction, and favorable stakeholder perception, businesses that engage in green projects are generally expected to have higher economic outcomes. As well as green investments can serve as an indicator of a company's passionate adoption of its social duty, which can enhance the company's public image and lead to better economic outcomes [62]. In light of the above arguments, we proposed that:

H1 Green Practices have a significant effect on economic performance

H1a Green Manufacturing Practice has a significant effect on economic performance

H1b Green Marketing orientation has a significant effect on economic performance

H1c Green HRM-training and development has a significant effect on economic performance

H1d Green investment has a significant effect on economic performance

### 2.3 Green practices and green innovation

#### 2.3.1 Green manufacturing and green innovation

Despite its early stages, the study of environmentally friendly innovations (GI) and green manufacturing practices (GMP) is drawing attention from academics, industry professionals, and policymakers [63]. Under GMP, businesses now give priority to developing innovative technologies, designing green products, and developing processes [64]. As an illustration [39], demonstrated that sustainable production techniques for the production of environmentally friendly products, several businesses face significant difficulties putting these techniques into effect across many industries. Likewise, Çankaya & Sezen [40], argued that green manufacturing techniques are a crucial component of improving environmentally friendly innovation-based capabilities to improve a firm's sustainability performance. It is asserted that environmental or green innovation is the pathway that leads to the impact of environmentally friendly production techniques on a business's sustainable outcome [65].

#### 2.3.2 Green marketing and green innovation

Researchers in the sustainability management community are increasingly curious about whether green marketing orientation positively affects eco-innovation given the idea that it enhances the innovative capabilities of businesses [44]. Market orientation helps businesses generate concepts for creative goods and services, which boosts their level of innovation and less market-oriented businesses may be headed to be minor inventive regarding introducing innovative offerings, which could lead customers to choose goods from rival businesses [66]. Sustainable marketing orientation is among the most effective strategies for a company to reach its innovative goals and also it is found to have a strong predictive power for value-based innovation in an investigation [67,68] among Ghanaian companies. Additionally, inferring from the foregoing debate, the study asserts that by focusing on green marketing, businesses can acquire pertinent information about changes in customer preferences and needs and can then appropriately respond by presenting green Value-driven and creative goods and services [67]. As well as environmental innovation provides an opportunity to significantly boost economic expansion. Businesses should concentrate on environmentally friendly innovations to prevent fines because it will help them use less energy and save money on waste handling and discharge [69]. On the contrary, a research study on 217 small and medium-sized businesses in Ghana was conducted by Afum et al. [67], showing that a focus on green marketing has no impact on environmentally friendly innovations.

#### 2.3.3 Green HRM and green innovation

A growing number of sustainability researchers, practitioners, and policymakers have directed their attention toward establishing the cause-and-effect relationship between environmental-oriented human resource management (HRM) and green innovations. For instance, research in 335 Saudi Arabian companies revealed that using green HR practices helps enterprises succeed in green innovation [54]. Similarly, Aftab et al. [55], asserted that environmentally conscious human resource management strategies and activities greatly boost sustainable innovation. Furthermore, the results of [26,70], and [71], demonstrate the beneficial effects of environmentally friendly HRM on fostering environmentally-friendly innovation. GHRM is required to promote innovation to increase it and produce a long-lasting competitive advantage. This evidence further supports the hypothesis that green innovation performance as well as greener HR procedures and approaches greatly boost green innovation from the viewpoint of academic staff [72]. Similarly, an investigation conducted by Shahzad et al. [73], across 316 Chinese manufacturing companies verified that green innovation is positively impacted by ecologically conscious HRM practices. This argument is consistent prior results of (Song et al., 2020). Moreover, companies may improve their green processes and product innovation by integrating environmental issues into their HRM -training and development practices [74].

#### 2.3.4 Green investment and green innovation

Findings from the research suggest that green innovations are substantially influenced thru green investments. For instance, Shuwaikh et al. [75], demonstrate that green innovation has a promising effect on a business's financial and environmental outcome when it comes to sustainability. Furthermore, technology innovations function as a mediating function in the link between eco-friendly investment and company green performance enhancement [76]. Academic studies have examined the influence of OFDI on environmentally conscious innovations and found that it encourages both unique and environmentally friendly forms of invention [61]. All of these results point to how important green investment is for promoting green innovation in businesses and the larger economy. Green investment is a major force behind green innovation since it finances clean technology R&D while supporting energy-efficient, energy-efficient, and environmentally friendly infrastructure initiatives [77]. Such financial arrival drives businesses and entrepreneurs to create novel solutions that transform the production and use of resources. An advantageous market atmosphere is also produced by green investment, which raises consumer demand for sustainable goods and services and encourages competition among companies to produce greener alternatives [78]. Furthermore, green investments create a positive feedback loop that draws in further funding when profitable and viable sustainable companies are shown by outstanding greener innovations [79]. Accordingly, we hypothesize it as.

H2 Green Practices have a significant effect on Green Innovation

H2a GMP has a significant effect on GIN

H2b GMO has a significant effect on GIN

H2c GHRM-training and development has a significant effect on GIN

H2d GIV has a significant effect on the GIN

### 2.4 Green innovation and economic performance

According to Aboelmaged & Hashem, and Nuryakin & Maryati [80,81], green innovation is currently acknowledged as an innovative company model which satisfies the rising need for reputable, eco-friendly goods that are rooted in environmental protection. Furthermore, green innovation reduces environmental harm by conserving energy efficiency, waste recycling, reducing pollution, utilizing environmentally friendly resources, and eliminating carbon emissions [82]. By lowering costs and gaining market share, green innovation may lessen adverse environmental effects while enhancing the economic outcome of the firms [82,83]. Eco-innovation aids businesses in obtaining a more effective and cost-effective use of their resources and lowering overall costs, strengthening their core competencies. Companies that use innovative strategies can achieve an assortment of competitive edge that boosts their productivity and profitability [84]. By putting green innovation management into practice, companies may grow market share, improve consumer loyalty, and keep more customers [85]. Being environmentally conscious may lead to chances for cost savings as well as access to specific markets, unique goods, and the sale of pollution-control equipment [86]. Green innovation is an intelligent decision that creates environmentally friendly goods and procedures to successfully balance financial success with sustainability [87]. Innovation in sustainable procedures and the introduction of ecologically conscious products are the two components of green innovation [11]. To minimize the harmful effects of rubbish on the ecology, eco-friendly product innovations seek to change product designs, remove hazardous elements, and choose more ecologically responsive materials. Moreover, eco-process innovation, conversely, seeks to recycle waste into commodities with a commercial value and lower energy consumption throughout the manufacturing process [11]. Green innovation is therefore the most effective way to protect the environment and generate financial gain. Drawing on the preceding discourse, the subsequent hypothesis is posited:

H3 Green innovation has a significant effect on Economic performance

### 2.5 Green innovation as mediator

This research looks at whether environmental innovation mediated the effect of GMP on a company's sustainability outcome or if GMP has a direct impact on it. Separate empirical research sheds light on how green inventions function as a facilitator in the relationship between sustainable manufacturing and financial success. To perform green manufacturing, industrial processes must incorporate sustainable processes and technology [88]. Green innovation can help firms distinguish from others in the market, achieve an edge over rivals, and attract new customer groups who value environmentally friendly goods [37]. In the words of Waheed et al. [65], sustainable manufacturing practices (GMP) are a foremost force behind eco-innovations (GIN) development and are capable of pursuing novel avenues for firms' sustainability performance [40]. When a company accepts and uses green resources for innovation, there is a relationship between GMP and Additionally, recent research has shown that GMP directly and favorably influences economic performance (EP) [38].

Similarly, scant literature indicated that eco-innovation facilitates the link between green HRM and firms' outcomes. For instance, Kanan et al. [74], asserted that among GHRM practices and firms’ sustainable performance is partly facilitated by green innovation. Furthermore, environmentally friendly innovation exhibits a substantial influence on environmental, social, and economic outcomes [54]. This also supports the recent finding of [89].

In the individual level analysis on the link between GMO and financial outcomes was conducted on the mediation function of environmentally conscious inventions. Negi et al. [44], for example, asserted that environmental marketing orientation has substantial consequences on environmentally conscious inventions. Likewise, green innovation has a substantial contribution to economic performance [90]. This outcome provides backing to the idea that combining green marketing orientation with green innovation results in improved economic performance [67].

Literature demonstrated that, for businesses aiming to attain sustainability and environmental protection, environmentally friendly invention has a beneficial influence on both environmental and financial outcomes [91]. Additionally, the link between economic and ecological performances is mediated by environmentally friendly inventions [75]. Furthermore, ecologically sound investing has a substantial influence on economic or sustainability performance and environmentally conscious inventions [92]. Additionally, environmentally conscious inventions have substantial consequences on economic outcomes [29]. These researchers, however, look at individual mediation. Green innovation must thus play the mediating function in conjunction with green behaviors and economic performance. Consequently, this study argues as follows:

H4 GIN mediates the relationship between GBP and EP

H4a GIN mediates the relation between GMP and EP

H4b GIN mediates the relationship between GMO and EP

H4c GIN mediates the relation between GHRM and EP

H4d GIN mediates the relation between GIV and EP

**Fig. 1:**
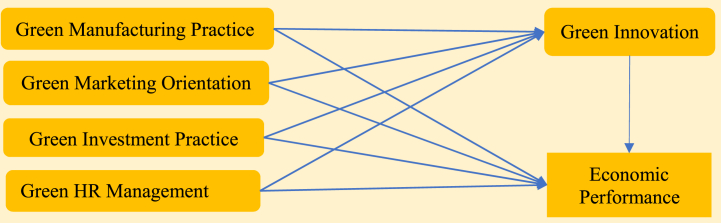
Research model.

The general framework of the relationship between the explanatory variables--green practices and outcome variable firms’ economic performance is exhibited in Fig. 1.

## 3 Material and methods

### 3.1 Study design

This investigation uses a quantitative methodology that consists of compiling and evaluating mathematical data to identify correlations, patterns, and trends across variables. In this instance, we obtained quantifiable information on institutional pressures, manufacturing businesses' performance, and green business practices.

### 3.2 Target population

To compile information for analysis, surveys were circulated to important participants in the survey, including general managers, executives, CSR team heads, and Environment department heads of medium- and large-sized leather, textile, and apparel companies. All the necessary procedures for a comprehensive census of Ethiopian manufacturing companies, characterizing the population according to size, location, and industry, were finished. Clear comprehension allowed for the identification of pertinent categories and the selection of participants by a random sampling method.

### 3.3 Sampling

The data gathering method used in the research made use of a random sample technique. Random sampling ensures that for each individual, there are equal opportunities for the population. To be chosen as a portion of the specimen. Afterward, methodical procedures were engaged in the random sample procedure in this specific study. The whole population of Ethiopia's medium- and large-scale leather, textile, and apparel manufacturing businesses was initially included in the extent of this investigation. A detailed list of these industries was then created, and each one was given a special code that was usually represented by a number. A spreadsheet tool's randomized number function was used to create a series of random numbers to add some element of chance to the process of selection. The next stage was comparing the numbers, which were produced at random, with the distinct IDs given to each industry. More specifically, research participants were chosen from among the industries that were connected to the linked codes. Out of 231, distributed using a stratified random sampling technique only 201 participants responded to this research, yielding an estimated response rate of 87 %.

### 3.4 Data collection

The research used a questionnaire with Likert scale items ranging from 1 (strongly disagree) to 5 (strongly agree) to gauge respondents' sentiments. This approach made it easier to gather quantitative data, which were then subjected to statistical analysis to find patterns and trends. The main source of data was questionnaire replies from medium- and large-sized Ethiopian companies that manufacture leather, textiles, and clothing. By concentrating on this industry, important insights were gained on the obstacles and prospects for introducing green business practices (GBP) to other sectors with major environmental effects. The use of surveys, which are well-known for being efficient in gathering quantitative data, was essential to this study.

### 3.5 Measurement

The survey was divided into three pieces. The backgrounds of the individuals were the main topic of the first session. The enterprise's profile information was collected in the second part, and thoughts regarding green business practices (GBP) and the financial performance of businesses were assessed in the last section. 39 elements in all, dispersed among six structures, are listed as follows: Six items measuring the green manufacturing (GMP) construct [38,93], a total of five items measuring GIV [94]; five items measuring GMO from literature [6,23], six items measuring GIN [38], six items measuring GHRM (training and development dimension alone) [49,95]; and five items measuring EP [37] (see appendix I).

### 3.6 Method of analysis

To explore complex interactions between observable and latent variables in a dataset, the current investigation employs structural equation modeling using partial least squares (PLS-SEM)), a statistical approach that combines factor analysis and regression [96]. Building a measurement model, assessing its validity and consistency, and building a path model to look into the association among unobserved variables are all part of the study. PLS-SEM is resilient to non-normal data distributions and can handle a range of sample sizes, making it ideal for exploratory research and theory building [97]. Additionally, it allows formative and reflective measurement methods to be included.

## 4 Result and discussion

### 4.1 Common method bias

The possible bias resulting from utilizing a single measurement approach for both independent and dependent variables is called common method bias, and it is examined in this study using the Inner and Outer Variance Inflation Factor (VIF) tests [98]. While the outer VIF test evaluates the collinearity between the measurement model and the structural model, identifying common technique bias and guaranteeing more accurate and dependable findings, the inner VIF test looks at the collinearity between constructs inside a measurement model. The result demonstrated that in Table 1, Table 6, the values of the outer (inner) VIF of each exogenous construct range from (1.181–1.897) < 3.3 criteria, as per the results (Table 1) [98]. As a result, there are no CMB or CMV issues.

**Table 1:** Inner model VIF.

Constructs	EP	GIN	Criteria (<3.3)
EP			
GHRM	1.181	1.178	Yes
GIN	1.796		Yes
GIV	1.451	1.406	Yes
GMP	1.389	1.363	Yes
GMO	1.897	1.453	Yes

### 4.2 Descriptive analysis

According to Table 2, which showed the respondents' gender distribution, 149 (74.1 %) of the respondents were men, and 73 (36.3 %) of the respondents had between seven and 10 years of expertise. Moreover, 102 (50.7 %) and 114 (56.7 %) of the responding enterprises were mostly textile companies. Furthermore, 128 respondents, or 63.7 % of the total, were general managers.

**Table 2:** Descriptive statistics.

Items	Options	Frequency	%
Gender	Male	149	74.1
	Female	52	25.9
Work experience	1–3 year	30	14.9
	4–6 years	67	33.3
	7–10 years	73	36.3
	Above 10 years	31	15.4
Firm category	Textile	102	50.7
	Leather	47	23.4
	Garment	52	25.9
Firm size	Medium size	114	56.7
	Large size	87	43.3
Level of respondent	General Manager	128	63.7
	CSR team head	59	29.4
	Environment and energy head	14	7.00

### 4.3 Measurement model

#### 4.3.1 Reliability and convergent validity

**Fig. 2:**
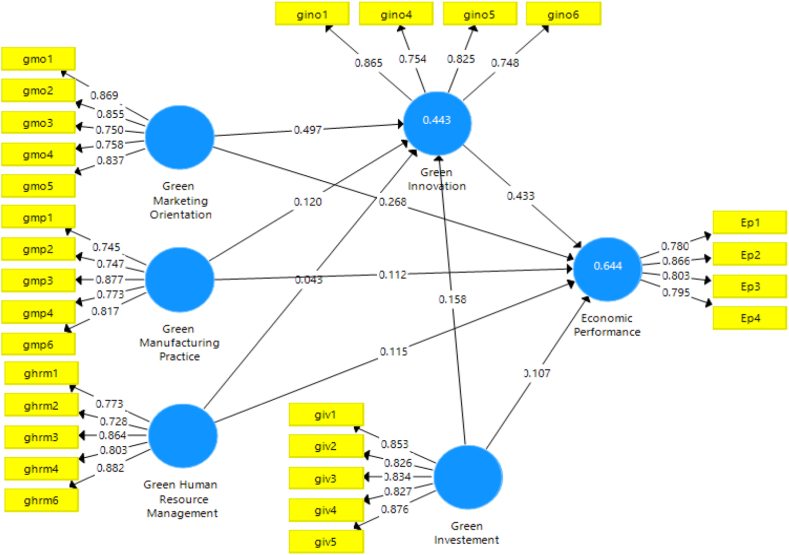
Factor loading.

Any variables included in this study had been examined for reliability as well as validity thru average variance extracted (AVE) and composite reliability (CR) [99]. The AVE values were more than the 0.5 value, while the CR values were higher than the suggested threshold of 0.70. The convergence validity was acceptable for all constructs. Any variable in AVE that has a square root greater than the correlations between any two constructs will have discriminative validity [100]. All constructs have sufficient, consistent, and discriminant validity, according to the results. Moreover, factor loading levels in the range of 0.70–0.90 are considered pleasing or good in exploratory studies [101]. Table 3 and Fig. 2, exhibited that the factor loading value ranges from 0.728 to 0.882 and Cronbach's alpha value ranges beginning between 0.810 and 0.899. As well as the AVE ranging from 0.629 to 0.712 which is within the acceptable threshold [96].

**Table 3:** Construct reliability and convergent validity.

Latent variables	Items	Factor Loadings	Cronbach's Alpha	rho_A	Composite Reliability	(AVE)
Economic Performance	Ep1	0.780				
	Ep2	0.866				
	Ep3	0.803	0.829	0.841	0.885	0.659
	Ep4	0.795				
Green human resource management	ghrm1	0.773				
	ghrm2	0.728				
	ghrm3	0.864	0.870	0.884	0.906	0.659
	ghrm4	0.803				
	ghrm6	0.882				
Green innovation	gino1	0.865				
	gino4	0.754				
	gino5	0.825	0.810	0.810	0.876	0.639
	gino6	0.748				
Green investment	giv1	0.853				
	giv2	0.826				
	giv3	0.834	0.899	0.903	0.925	0.712
	giv4	0.827				
	giv5	0.876				
Green marketing orientation	gmo1	0.869				
	gmo2	0.855				
	gmo3	0.750	0.878	0.912	0.908	0.665
	gmo4	0.758				
	gmo5	0.837				
Green manufacturing practices	gmp1	0.745				
	gmp2	0.747				
	gmp3	0.877	0.852	0.864	0.894	0.629
	gmp4	0.773				
	gmp6	0.817				

Note: item gino2, gino3, gmp5, ghrm5 was removed (loading <0.70).

#### 4.3.2 Discriminant validity

Building upon construct validity is indispensable to confirm that any concept in the research is distinct from the other constructs [102]. As a result, we use the Hetrotriat-Monotriant-ratio (HTMT) test, the Fornell-Larcker criteria, and cross-loadings to examine discriminating relevance [97]. When HTMT values are above the recommended cut-off point of 0.85–0.90 for structural models with conceptually relatively identical components, discriminant validity issues arise [97]. The discriminant validity of the research as illustrated in Table 4, Table 5, Table 6 has been proven.

**Table 4:** Discriminant validity (Fornell-Larker criteria).

Variables	EP	GHRM	GIN	GIV	GMP	GMO
Economic Performance	0.812					
Green Human Resource Mgt	0.388	0.812				
Green innovation	0.727	0.290	0.799			
Green investment	0.487	0.196	0.438	0.844		
Green Manufacturing	0.467	0.221	0.401	0.463	0.793	
Green Marketing orientation	0.675	0.381	0.630	0.434	0.398	0.816

**Table 5:** Discriminant validity (cross loading).

Items	EP	GHRM	GIN	GIV	GMP	GMO	VIF
Ep1	0.780	0.266	0.672	0.418	0.381	0.580	1.909
Ep2	0.866	0.278	0.722	0.448	0.437	0.661	2.269
Ep3	0.803	0.337	0.450	0.366	0.351	0.452	3.197
Ep4	0.795	0.406	0.456	0.331	0.331	0.457	3.212
ghrm1	0.264	0.773	0.208	0.153	0.106	0.276	3.679
ghrm2	0.282	0.728	0.234	0.184	0.225	0.357	1.565
ghrm3	0.362	0.864	0.228	0.132	0.222	0.289	3.997
ghrm4	0.259	0.803	0.230	0.148	0.133	0.279	3.939
ghrm6	0.383	0.882	0.274	0.179	0.196	0.343	4.292
gino1	0.562	0.219	0.865	0.374	0.348	0.521	4.028
gino4	0.604	0.256	0.754	0.342	0.362	0.476	1.472
gino5	0.519	0.186	0.825	0.304	0.266	0.490	3.702
gino6	0.622	0.258	0.748	0.371	0.296	0.519	1.440
giv1	0.456	0.141	0.338	0.853	0.359	0.390	3.148
giv2	0.389	0.226	0.368	0.826	0.429	0.376	2.427
giv3	0.363	0.117	0.355	0.834	0.375	0.313	2.952
giv4	0.378	0.190	0.369	0.827	0.347	0.345	2.454
giv5	0.459	0.155	0.415	0.876	0.438	0.401	2.617
gmp1	0.334	0.124	0.346	0.437	0.745	0.336	1.590
gmp2	0.300	0.157	0.244	0.312	0.747	0.253	2.350
gmp3	0.442	0.240	0.346	0.417	0.877	0.355	3.321
gmp4	0.352	0.203	0.265	0.335	0.773	0.296	2.544
gmp6	0.401	0.148	0.366	0.328	0.817	0.326	2.627
gmo1	0.508	0.289	0.495	0.345	0.310	0.869	3.997
gmo2	0.484	0.295	0.444	0.357	0.339	0.855	3.967
gmo3	0.381	0.366	0.356	0.213	0.171	0.750	2.712
gmo4	0.758	0.262	0.708	0.465	0.446	0.758	1.387
gmo5	0.445	0.380	0.397	0.282	0.240	0.837	3.567

**Table 6:** Discriminant validity (HTMT).

Variables	EP	GHRM	GIN	GIV	GMP	GMO
Economic Performance						
Green Human resource mgt	0.459					
Green innovation	0.859	0.342				
Green investment	0.554	0.223	0.510			
Green Manufacturing	0.543	0.251	0.473	0.527		
Green Marketing orientation	0.724	0.444	0.693	0.455	0.422	

#### 4.3.3 Model fit

For the reason that the PLS-SEM technique is designed to maximize explained variance, the literature cautions against placing too much reliance on global model fit indices [103]. Rather, it assesses the explanatory and predictive capacities of the model. Predictive relevance (Q^2^), effect size (F^2^), and coefficient of determination (R^2^) are the main metrics used to evaluate model fit [104]. We evaluated the structural model for model fit in PLS-SEM, by measuring the coefficient of determination (R^2^) and effect size (F^2^). The result in Table 7 exhibited that, *R*^2^ = 0.443 and 0.644, respectively, for the endogenous constructs GIN and EP confirming a reasonable/moderate predictive accuracy [104]. GIN is improved by 44.3 % and EP is improved by 64.4 % when GMP, GMO, GHRM, and GIV are integrated. Following evaluating coefficients of determination, the study employed the blindfolding approach at omission distance case seven to evaluate the predicting significance (*Q*^2^), the *Q*^2^ result in Table 7 illustrated that the exogenous constructs of GIN and EP, (0.265 and 0.399), respectively, was observed this indicate distinct from zero and consistent [104]. Cohen [105] recommended interpreting effect size in exploratory and predictive studies using the f-squared (f2) statistic. F2 ≥ 0.02, f2 ≥ 0.15, and f2 ≥ 0.35 denote small, medium, or large impacts, respectively. As Table 7 illustrates the score of the f-squared (f2) statistic ranges from small (0.003 to large impact 0.293) [105].

**Table 7:** Coefficient of determination (R^2^), and predictive relevance (Q^2^) and effect size (F^2^).

Exogenous Constructs	F^2^		(R^2^),	R^2^ Adjusted	(Q^2^)
	EP	GIN			
Economic Performance			0.644	0.635	0.399
Green Innovation	0.293		0.443	0.432	0.265
Green HRM	0.031	0.003			
Green Investment	0.022	0.032			
Green Manufacturing Practice	0.025	0.019			
Green Marketing Orientation	0.106	0.305			

### 4.4 Structural model

Following the examination of the measuring model, we move on to the structural analysis phase to get a deeper understanding [96]. That underlies the links between sustainable business practices and economic outcomes. This stage of the PLS-SEM analysis enables us to test our models and present actual data [97] regarding how sustainable practices contribute to improving economic outcomes. Therefore, to determine the significance of each structural route, a bootstrapping method was used in this study.

Green manufacturing is expected to improve firm economic performance (EP), according to the first hypothesis (H1a). Our findings revealed a noteworthy association between green manufacturing and a business's economic outcome. The investigation revealed a scientifically substantial link between GMP and EP, as illustrated by the beta coefficient (β = 0.112, t = 2.375, p = 0.018). Thus, the study backs up H1a, which asserts that using green manufacturing techniques improves a company's financial success.

**Fig. 3:**
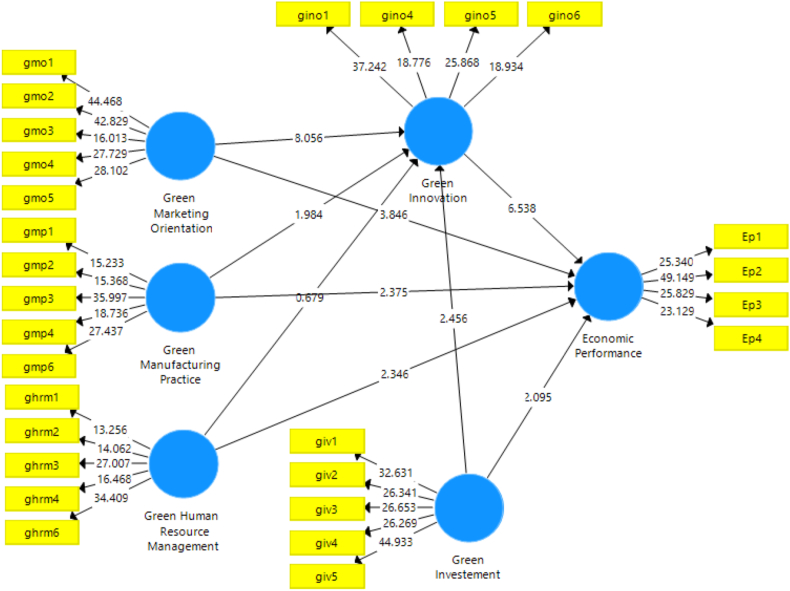
Bootstrapping result (5000).

This study also examines the second hypothesis (H1b), green marketing orientation (GMO) has a notable impact on firms’ EP. Findings from the examination illustrate that GMO substantially and favorably affects the economic performance of businesses, as shown in Fig. 3 and Table 8 (β = 0.268, t = 3.846, p = 0.000). Accordingly, H1a is supported by this study.

**Table 8:** Hypothesis test (direct effect).

Hypothesis	Relationship	Beta (β)	St. Dev	t- Statistics	P- Values	Support?
H1c	GHRM - > EP	0.115	0.049	2.346	0.019***	Yes
H2c	GHRM - > GIN	0.043	0.064	0.679	0.497	No
H3	GIN - > EP	0.433	0.066	6.538	0.000***	Yes
H1d	GIV - > EP	0.107	0.051	2.095	0.036***	Yes
H2d	GIV - > GIN	0.158	0.065	2.456	0.014***	Yes
H1a	GMP - > EP	0.112	0.047	2.375	0.018***	Yes
H2a	GMP - > GIN	0.120	0.060	1.984	0.047***	Yes
H1b	GMO - > EP	0.268	0.070	3.846	0.000***	Yes
H2b	GMO - > GIN	0.497	0.062	8.056	0.000***	Yes

Note: ***are significant at 0.05.

Furthermore, we look at how important GHR practices are to improve the company's economic results, particularly the ecological education and development aspect (H1c). Our findings confirm hypothesis three by showing that green HR practices statistically substantially affect the firm's EP (β = 0.155, t = 2.346, p = 0.019). In addition, the result in Table 8 and Fig. 3 confirms H1d by showing that green investment (GIV) has a substantial effect on EP (β = 0.107, t = 2.095, p = 0.036). We also accept hypothesis H3 since Table 8 shows that green innovation (GIN) considerably improves enterprises' EP (β = 0.433, t = 6.538, p = 0.000).

Similarly, we investigate how green business practices directly affect the mediating variable (GIN). Specifically, the interaction between GIN and GMP, GMO, GHRM, and GIV. GMP substantially and favorable effect GIN (β = 0.120, t = 1.984, p = 0.047), supporting H2a, according to the results in Table 8 and Fig. 3. This study validates H2b and reveals the significant role that GMOs had in the evolution of GIN (β = 0.497, t = 8.056, p = 0.000). Similarly, H2d is affirmed with our discovery of the direct impact of GIV on GIN, which shows that GIV significantly affects GIN (β = 0.158, t = 2.456, p = 0.014). The impact of GHRM on GIN in this investigation is negligible (β = 0.043, t = 0.679, p = 0.497). As a result, it does not support H2c (see Table 8 and Fig. 3).

### 4.5 Mediation effect

To look into the possible interceding function of GIN in the link among sustainable manufacturing, green investment, green marketing orientation, green HRM, and businesses' financial outcomes, we run a mediation study. Table 9 shows a statistically significant relationship between green marketing attitude and economic success (H4b: β = 0.215, t = 4.990, p = 0.000). Even after adjusting for a mediating variable (GIN), the link between environmentally friendly investment and companies' economic performance (EP) remained (β = 0.069, t = 2.306, p = 0.021). Nonetheless, the mediated impact of ecologically responsible manufacturing practices (GMP) on business economic outcomes through green innovation (GIN) was statistically insignificant (β = 0.052, t = 1.858, p = 0.063). Furthermore, H4a is not supported because of the insignificant mediating effect of GIN on the link between GHRM, more especially green training, and development, and economic outcome (β = 0.019, t = 0.670, p = 0.503).

**Table 9:** Mediation (indirect effect).

Hypothesis	Relationship	β	St. Dev.	t- Statistics	P- Values	Support?
H4c	GHRM - > GIN - > EP	0.019	0.028	0.670	0.503	No
H4d	GIV - > GIN - > EP	0.069	0.030	2.306	0.021	Yes
H4a	GMP - > GIN - > EP	0.052	0.028	1.858	0.063	No
H4b	GMO - > GIN - > EP	0.215	0.043	4.990	0.000	Yes

## 5 Discussion

This study looks at how environmentally friendly practices, such as green manufacturing practices (GMP), green investment (GIV), green HRM, and green marketing orientation (GMO), affect Ethiopian leather and textile companies' economic performance (EP). Additionally, take a look at the function of sustainable innovations (GIN) as a mediator in this connection. As a consequence, the findings shed fresh light on how implementing green practices affects a company's financial success in addition to the moderating impact of ecologically conscious invention.

Our first finding discloses that GMP has a noteworthy impact on economic performance (EP) as proposed in H1a. This implied that businesses should adopt environmentally friendly manufacturing practices if they look to improve their financial results. Additionally, eco-friendly manufacturing techniques like waste minimization and resource efficiency can lead to reduced expenses and better production methods, which eventually boost financial performance [106]. This finding is consistent with [37,107] which underscores the significance of environmentally friendly production techniques in boosting financial success. However, this is also contradictory to Refs. [11,108] who asserted that green manufacturing has no positive contribution to a firm's economic performance (EP).

Secondly, we find out that green marketing orientation (GMO) has a substantial contribution in boosting the firm's economic performance H1b. This supports the prior findings of [23,44,109]. This means that a focus on green marketing, which includes endorsing and providing eco-friendly goods and services, may draw in eco-aware customers and grow market share and income.

The third finding of the study in Table 8 demonstrates that GHRM practices significantly and favorably affect financial performance (H1c). This is consistent with [48,54,110]. The effect of GHRM training and development, which comprises programs like education and development centered on sustainability and environmental awareness, may improve employees' knowledge and abilities, which will increase productivity and operational efficiency this in turn improves the firm's economic performance [111].

Additionally, our result about the effect of GIV on firms’ EP (H1d) exhibited in Table 8 demonstrated that green investment strategies, such as funding sustainable technologies and R&D projects, may spur innovation, boost competitiveness, and create long-term value, all of which have a beneficial contribution to EP. This also support the prior finding of [62,77,92]. However, this finding, in contrast to Ref. [16], asserts that investment in eco-friendly activities will drain firms' finances and be considered a cost for firms.

Moreover, our conclusion revealed that green innovation has a noteworthy impact on economic outcomes. This is consistent with [37,86,112]. Accordingly, companies looking to increase their profitability should adopt environmentally friendly procedures and engage in product innovation, since these will ultimately improve their financial results.

Our analysis shows a substantial association between environmentally sustainable practices and eco-innovation, specifically, green manufacturing practice (GMP) and green innovation (GIN) this supports the findings of [38,63]. Additionally, green investment substantially supports the creation of environmentally sustainable products and processes which is consistent with [75,77], and green marketing orientation having the most impact on the organizations' degree of green innovation [44], and [113]. According to this research, implementing these principles may inspire creative initiatives for environmental stewardship and sustainability. Enterprises may augment their capacity to create in harmony with green principles by incorporating environmentally conscious processes, creating new sustainable goods, and integrating eco-friendly technology.

Notably, though, is that green innovation was not much impacted by the green HRM component—that is, green training and development [54]. This contradicts [114], and [115] findings. Furthermore, green practices, and other elements like company culture, leadership backing, and outside partnerships could be more important in fostering innovation.

Regarding green innovation's mediating function, the findings indicate that it modifies, to a certain extent, the link between sustainable investment, and economic success. Our finding is conformable to Ref. [78], and [116] findings. Furthermore, the intervening function of GIN in the link between green marketing orientation, and EP was supported H4d. This data suggests that the way these behaviors affect economic performance is through a process known as eco-friendly innovations. It is correspondent to Refs. [44,82] conclusion. This implies that firms engage in green marketing orientation and simultaneously improve their green process and product innovation, which in turn boosts economic performance. Businesses may create new eco-friendly goods, adopt sustainable practices, and investigate market potential by promoting green innovation, all of which can enhance their financial performance.

Nonetheless, the findings suggest that green innovation does not mediate the link between green manufacturing, and economic success (H4a). This is in contrast with [37,108]. This implies that processes other than green innovation may be involved in the way these practices affect economic performance. Moreover, green innovation does not mediate the association between green HRM practice and economic performance H4c. This is in contrast with [117] who confirmed that engaging in green training and development amends employees to be ecologically aware and it causes improved productivity and economic achievement.

## 6 Conclusion

This research investigated the effects of green practices on the economic performance (EP) of companies in Ethiopia's leather, textile, and apparel sectors. More precisely, the study focused on the effects of green manufacturing practices (GMP), green marketing orientation (GMO), green investment (GIV), and sustainable human resources management (GHRM training and development). The findings show that green innovation (GIN) and the financial performance of businesses are significantly enhanced by GMP, GMO, and GIV. However, the use of GHRM training and development procedures does not significantly impact the advancement of GIN.

Additionally, the results show that GIN mediates the interaction between EP and both GIV and GMO. This implies that the main way that green innovation drives a firm's economic performance is through investments in green projects and the adoption of a green marketing approach. By contrast, the relationship between GHRM, GMP, and economic performance is not mediated by GIN.

These findings highlight how crucial green practices—in particular, GMP, GMO, and GIV—are to the financial success of Ethiopian leather, textile, and apparel companies as well as to environmental sustainability. The results underscore the necessity for companies operating in these sectors to give top priority to implementing environmentally friendly policies that foster green innovation, as this seems to be a crucial pathway via which these practices lead to better economic results.

In general, this research offers significant perspectives for managers and policymakers who seek to advance the economic and ecological sustainability of Ethiopia's leather, textile, and apparel industries. The findings point to particular green practices that businesses should concentrate on putting into place to improve their financial performance by encouraging green innovation.

### 6.1 Practical Implications

Companies' financial performance could be greatly enhanced by green business practices, including green investing, green production, and green marketing strategies. These methods may result in lower expenses, more effective operations, enhanced brand recognition, and the opening up of new market niches. Green training and development are crucial for raising employee awareness and understanding of sustainability even if it might not immediately lead to green innovation. Companies should keep funding environmentally conscious training and development initiatives to foster an organizational culture of environmental stewardship.

### 6.2 Policy Implications

The governments and oversight organizations ought to implement measures, such as tax breaks, subsidies as well as grants, to encourage and facilitate the application of eco-friendly corporate activities. These actions can incentivize companies to spend money on environmentally friendly production, marketing, and innovation, which will benefit the economy and the environment. To ease the exchange of best practices in the implementation of green business strategies, policymakers should think about encouraging collaboration and knowledge sharing across enterprises. This has the potential to quicken industry adoption of sustainable practices.

### 6.3 Implication to achieve UN SDGs

This study investigates the link between Ethiopian leather and textile companies' financial success and green company practices, including green marketing, green production, green human resource management, and green investment. It draws attention to how crucial green innovation is to fostering sustainable corporate practices, increasing employment, and fostering long-term success. The result of this study can serve as a roadmap for Ethiopian businesses, governments, and stakeholders to advance sustainable economic growth and decent work, and foster green innovation and environmentally friendly business practices thus supporting Sustainable Development Goals 8 and 9.

### 6.4 Future research directions

The underlying elements that prohibit green training and development from having a direct influence on green innovation require more investigation. Comprehending the fundamental processes might assist in customizing educational initiatives to enhance sustainable innovation. A thorough comprehension of the processes underlying the link among green business practices and economic performance may be obtained by looking at additional possible moderators or mediators. The long-term impact of green business practices on companies' financial performance, besides the durability of these benefits over time, may be evaluated through longitudinal research. The context-specific elements that affect the efficacy of green business practices across various sectors, firm sizes, and regions might be the subject of future research. Analyzing the perceptions and behaviors of consumers toward environmentally friendly goods and services can provide additional insight into market trends and the financial performance of environmentally friendly innovations in businesses.

## Data availability statement

The dataset that supports this study's findings is available upon reasonable request.

## Ethics approval and consent to participant

The authors of this work adhere to the strictest ethical guidelines while researching willing human subjects. To commence the research activities, the authors have received ethical letters from the Arba Minch University School of Graduate Studies of Doctoral Programs Coordination Office (*with Ref No. Mgmt/109/16*). The study complied with the guidelines for minimizing harm, informed consent, data security, and confidentiality. The submission of the paper certifies that the study was carried out following all relevant ethical standards and laws.

The verbal agreement was acquired from participants voluntarily and openly before the data collection procedures of this study. The authors explained to the participants that the collected data were merely utilized for academic reasons and that no fees were offered to the research subjects (participants). The authors also explained verbally to the study participants how participant anonymity was safeguarded, how data collection was done, the ability to request questions, the freedom to revoke consent at any time, the lack of penalty for doing so, and the research benefits. Finally, the participants verbally granted the author's informed consent.

## Funding

Not applicable.

## CRediT authorship contribution statement

**Tilahun Nigatu:** Writing – original draft, Validation, Software, Methodology, Investigation, Formal analysis, Data curation, Conceptualization. **Aschalew Degoma:** Writing – review & editing, Visualization, Validation, Supervision, Project administration, Methodology, Conceptualization. **Abiot Tsegaye:** Writing – review & editing, Validation, Supervision, Software, Project administration, Methodology, Conceptualization.

## Declaration of competing interest

The authors declare that they have no known competing financial interests or personal relationships that could have appeared to influence the work reported in this paper.

## Figures and Tables

**Table:** 

Code	Measurements/Items
**Green manufacturing practice (GMP)**	
gmp1	Our company evaluates the environmental load of products using life-cycle assessment.
gmp2	Our company produces textiles with reused and recycled contents.
gmp3	Our company reduces power consumption during manufacturing and transportation.
gmp4	Our company produces textiles/leather that are free from hazardous substances.
gmp5	Our company uses eco-friendly technology and equipment during the production process.
gmp6	Our company reduces the consumption of materials to produce textiles.
**Green Marketing Orientation**	
gmo1	Our company conducts market research to identify green needs in the marketplace.
gmo2	Our company supports the practice of e-commerce, as it is more eco-friendly.
gmo3	Our company favors digital communication methods for promoting our products, as it is eco-friendly.
gmo4	Our company practices a paperless policy in our procurement where possible.
gmo5	Our company attempts to use renewable energy sources for our products/services.
gmo6	Our company carefully monitors the level of commitment to serving customers' environmental needs.
**Green Innovation (GI)**	
gino1	We select the materials of the product that produce the least amount of pollution for conducting the product development or design.
gino2	We select the materials of the product that consume a minimum amount of energy and resources in conducting the product development or design.
gino3	We utilize the fewest amounts of materials to comprise the product in conducting the product development or design.
gino4	We cautiously consider whether the product is easy to recycle, reuse, and decompose in conducting the product development.
gino5	Our company's production process successfully reduces the release of hazardous substances or waste.
gino6	Our company's production process decreases water energy and raw materials consumption.
**Green Investment (GIV)**	
giv1	Our company finances product research and development toward the green direction.
giv2	Our company finances renewable energy systems.
giv3	Our company finances wastewater treatment systems according to international standards.
giv4	Our company finances exhaust gas treatment systems according to international standards.
giv5	Our company finances green technology applications.
**Green HRM (training and development)**	
ghrm1	Our Firm provides conservational training programs on working methods to reduce environmental degradation.
ghrm 2	Our Firm develops training programs in environment management to increase employee's environmental awareness.
ghrm 3	Our Firm gives ecological training to employees such as reducing waste, recycling, and saving electricity.
ghrm 4	Our Firm evaluates in which green aspects the employee needs training.
ghrm 5	Our Firm evaluates who needs training in environmental management.
ghrm 6	Our Firm engages employees in green events and assists them in environmental management learning.
**Economic performance (EP)**	
Ep1	In our company, there are improved profits
Ep2	In our company, there is sales growth.
Ep3	In our company, there are good returns on investment.
Ep4	In our company, there are good returns on equity.
Ep5	In our company, there are good returns on assets.
