# Supplementary material for: Green practices and economic performance: Mediating role of green innovation in Ethiopian leather, textile, and garment industries—An integrated PLS-SEM analysis
Source: Heliyon. 2024 Jul 25;10(15):e35188. doi: 10.1016/j.heliyon.2024.e35188 (PMC11328105; doi:10.1016/j.heliyon.2024.e35188)
Supplement: Multimedia component 1 [file mmc1.docx]

**Appendix**

**ARBA MINCH UNIVERSITY**

**College of Business and Economics**

**Department of Management**

Dear respondents, I am currently a Ph.D. Candidate in management studies at Arba Minch University department of Management. As a partial fulfillment, I am conducting my Dissertation entitled *“The Effect Green Business practices on sustainable Business performance: Through Green Innovation in the case of Ethiopian Leather, Textile and Garment firms”.* Therefore, I would appreciate for your spare a few minutes regarding the following questions in green marketing, green manufacturing, green investment, green dynamic capability and green innovation practices’ influence on company performance (social, economic, and environmental) in your organization.

So, you are kindly requested to provide accurate, complete topic-related reliable information to the best of your knowledge. Feel free to answer any topic-related issues since the data will be used for this specific research or academic purpose only. I assure you that all the information will be kept confidential.

**Thank you in advance for your cooperation and timely response**!

**General instruction**: Put a Tick Mark **(✓)**

**Part I: Demographic information**

1. Gender: male ( ) Female ( )
2. Year of work experience in the organization

1-3 year ( ) 4-6 years ( ) 7-10 years ( ) above 10 years ( )

1. Your current position in the organization

General manager ( ) Environment and energy head ( )

Corporate social responsibility CSR head (

1. Firm category

Leather ( ) Textile ( ) Garment ( )

1. Firm size: Medium size ( ) Large size ( )

**Part II: Green business practices and sustainability performance related questions**

Please put **Tick Mark (✓)** on your response to indicate your level of agreement from the questions given related to green business practices.

| **code** | **Scale: 1 = Strongly Disagree, 2 = Disagree, 3 = Neutral, 4 =Agree, and 5 = Strongly agree**. | **1** | **2** | **3** | **4** | **5** |
| --- | --- | --- | --- | --- | --- | --- |
|  | **Green manufacturing practice (GMP)** |  |  |  |  |  |
| gmp1 | Our company evaluate the environmental load of products using life-cycle assessment |  |  |  |  |  |
| gmp2 | Our company produces textile with reused and recycled contents |  |  |  |  |  |
| gmp3 | Our company reduces power consumption during manufacturing and transportation |  |  |  |  |  |
| gmp4 | Our company produces textile/leather that are free from hazardous substances |  |  |  |  |  |
| gmp5 | Our company use eco-friendly technology and equipment during production process |  |  |  |  |  |
| gmp6 | Our company reduce the consumption of materials to produces textiles |  |  |  |  |  |
|  | **Green Marketing Orientation** |  |  |  |  |  |
| Gmo1 | Our company conduct market research to identify green needs in the marketplace |  |  |  |  |  |
| Gmo2 | Our company support the practice of e-commerce, as it is more eco-friendly |  |  |  |  |  |
| Gmo3 | Our company favor digital communication methods for promoting our products, as it is eco-friendly |  |  |  |  |  |
| Gmo4 | Our company practice a paperless policy in our procurement where possible |  |  |  |  |  |
| Gmo5 | Our company attempt to use renewable energy sources for our products/services |  |  |  |  |  |
| Gmo6 | Our company carefully monitor the level of commitment in serving customer environmental needs |  |  |  |  |  |
|  | **Green Innovation (GI)** |  |  |  |  |  |
| gino1 | We select the materials of the product that produce the least amount of pollution for conducting the product development or design |  |  |  |  |  |
| gino2 | We select the materials of the product that consume a minimum amount of energy and resources in conducting the product development or design. |  |  |  |  |  |
| gino3 | We utilize the fewest amounts of materials to comprise the product in conducting the product development or design. |  |  |  |  |  |
| gino4 | We cautiously consider whether the product is easy to recycle, reuse, and decompose in conducting the product development |  |  |  |  |  |
| gino5 | Our company production process successfully reduces the release of hazardous substances or waste |  |  |  |  |  |
| gino6 | Our company production process decreases water energy and raw materials consumption |  |  |  |  |  |
|  | **Green Investment (GIV)** |  |  |  |  |  |
| Giv1 | Our company finances product research and development toward the green direction |  |  |  |  |  |
| Giv2 | Our company finances renewable energy systems |  |  |  |  |  |
| Giv3 | Our company finances wastewater treatment systems according to international standards |  |  |  |  |  |
| Giv4 | Our company finances exhaust gas treatment systems according to international standards |  |  |  |  |  |
| Giv5 | Our company finances green technology applications |  |  |  |  |  |

| Code | **Scale: 1 = Strongly Disagree, 2 = Disagree, 3 = Neutral, 4 =Agree, and 5 = Strongly agree** | **1** | **2** | **3** | **4** | **5** |
| --- | --- | --- | --- | --- | --- | --- |
|  | **Green HRM (training and development)** |  |  |  |  |  |
| ghrm1 | Our Firm provides conservational training programs on working methods to reduce environmental degradation |  |  |  |  |  |
| ghrm 2 | Our Firm’s develops training programs in environment management to increase employee’s environmental awareness |  |  |  |  |  |
| ghrm 3 | Our Firm’s gives ecological training to employees such as reducing waste, recycling, saving electricity |  |  |  |  |  |
| ghrm 4 | Our Firm’s evaluates in which green aspects the employee need training. |  |  |  |  |  |
| ghrm 5 | Our Firm’s evaluates who need training in environmental management |  |  |  |  |  |
| ghrm 6 | Our Firm’s engage employees in green events and assist them in environmental management learning |  |  |  |  |  |
|  | **Economic performance (EP)** |  |  |  |  |  |
| Ep1 | In our company there are improved profits |  |  |  |  |  |
| Ep2 | In our company there are sales growth |  |  |  |  |  |
| Ep3 | In our company there are good returns on investment |  |  |  |  |  |
| Ep4 | In our company there are good returns on equity |  |  |  |  |  |
| Ep5 | In our company there are good returns on asset |  |  |  |  |  |

**Thank you!**
